# Integrated management of neonatal and childhood illness strategy in Zimbabwe: An evaluation

**DOI:** 10.1371/journal.pgph.0000046

**Published:** 2021-11-22

**Authors:** Nigel James, Yubraj Acharya

**Affiliations:** Department of Health Policy and Administration, The Pennsylvania State University, University Park, State College, Pennsylvania, United States of America; Harvard University T H Chan School of Public Health, UNITED STATES

## Abstract

More than five million children under the age of five die each year worldwide, primarily from preventable and treatable causes. In response, the World Health Organization’s Integrated Management of Childhood Illnesses (IMNCI) strategy has been adopted in more than 95 low- and middle-income countries, 41 of them from Africa. Despite IMNCI’s widespread implementation, evidence on its impact on child mortality and institutional deliveries is limited. This study examined the effect of IMNCI strategy in the context of Zimbabwe, where neonatal and infant mortality rates are among the highest in the world. We used binary logistic regression to analyze cross-sectional data from the 2015 Zimbabwe Demographic and Health Survey. Zimbabwe implemented the IMNCI strategy in 2012. Our empirical strategy involved comparing neonatal and infant mortality and institutional deliveries within the same geographic area before and after IMNCI implementation in a nationally representative sample of children born between 2010 and 2015. Exposure to IMNCI was significantly associated with a reduction in neonatal mortality (adjusted odds ratio (95% CI): 0.70 (0.50, 0.98)) and infant mortality (adjusted odds ratio (95% CI): 0.69 (0.54, 0.91)). The strategy also helped increase institutional deliveries significantly (adjusted odds ratio (95% CI): 1.95 (1.67, 2.28)). Further analyses revealed that these associations were concentrated among educated women and in rural areas.The IMNCI strategy in Zimbabwe seems to be successful in delivering its intended goals. Future programmatic and policy efforts should target women with low education and those residing in urban areas. Furthermore, sustaining the positive impact and achieving the child health-related Sustainable Development Goals will require continued political will in raising domestic financial investments to ensure the sustainability of maternal and child health programs.

## Introduction

Low- and middle-income countries (LMICs) have made substantial progress in reducing child mortality in recent decades [1]. However, the reduction rates have not been uniform across different child age groups and geographic regions. Most deaths are concentrated among the neonates—those in the first 28 days of life—and predominantly occur in West and Central Africa, sub-Saharan Africa, and south Asia [2–4]. A UNICEF global study reported that sub-Saharan Africa had the highest neonatal mortality rate in 2018, at 28 deaths per 1000 live births, followed by Central and Southern Asia with 25 deaths per 1000 live births. Infant mortality—death occurring before the age of 12 months—is the highest in West and Central Africa, with 73 deaths per 1000 live births, followed by sub-Saharan Africa and Southern Asia, with 58 deaths and 36 deaths per 1000 live births, respectively [5]. Most neonatal deaths occur due to preventable or treatable conditions such as acute respiratory infections, diarrhea, measles, malaria, and malnutrition (WHO, 2017). Globally, neonatal mortality should drop from 27.8 million to 22.7 million by 2030 to achieve the Sustainable Development Goal (SDG) target of 12 deaths per 1000 live births [4]. Relatedly, more than 60 countries need to accelerate their progress from the current pace in order to reach this goal [4, 6].

In an effort to improve child survival, in the mid-1990s, the World Health Organization (WHO) devised a strategy called the Integrated Management of Childhood Illnesses (IMCI) which 95 countries, 41 of them in Africa, are currently implementing. In recent years, several countries are expanding the IMCI strategy to orient focus on neonatal deaths, as these deaths have remained stubbornly high. The new strategy is referred to as Integrated Management of Neonatal and Childhood Illnesses (IMNCI).

IMNCI strategy aims to improve child survival and development through three main pillars: (i) training of health workers on improved diagnosis and treatment measures; (ii) health systems strengthening for child health services delivery including adequate stocking of drugs, supervision, and enhanced monitoring and evaluation; and (iii) community and household interventions that address predisposing factors to childhood illnesses [7, 8]. In the first pillar, healthcare workers, including doctors, nurses and auxiliary staff, receive either six or eleven days of pre-service or in-service training on clinical management of childhood illnesses. The training focuses on a holistic diagnostic approach that utilizes cost-effective clinical management tools and protocols [9, 10]. The second pillar deals with efficient and effective supply chain management of drugs and medicines. Facilities are equipped with technical expertise and resources to facilitate improved health information systems for disease classification, data collection, reporting, and electronic decision support systems [11]. The third pillar focuses on providing education and counselling to mothers and/or caregivers on areas such as exclusive breastfeeding, complementary feeding, home hygiene, immunizations, and timely health care seeking.

This child survival strategy follows a broad implementation arrangement with multiple child health impact pathways. It mostly works through reductions in deaths from five causes: pneumonia, diarrhea, malaria, measles, and malnutrition. Countries that are implementing all the three pillars are classified as “full implementers.” Examples include Uganda, Tanzania, Zimbabwe, India, Bangladesh, Brazil, and Peru [7]. By 2016, more than 95 WHO member countries were implementing IMNCI at a large scale [12].

Despite IMNCI’s significance for virtually all LMICs, there is limited, albeit emerging, empirical evidence on its effects on neonatal mortality [12]. According to the WHO’s impact evaluation model, child mortality is an important impact indicator in evaluating IMNCI [7]. However, most countries’ evaluations so far assess process-related indicators such as adherence to treatment protocol, quality of care, vaccination coverages, exclusive breastfeeding, and patient satisfaction. For example, a study based on Tanzania reported a 63% vs 38% treatment protocol adherence rates between IMCI intervention and control sites [13]. Similar trends for improved clinical quality of care have been reported in India, Bangladesh, and Zimbabwe [14–16]. However, some researchers have warned against looking at IMNCI as a silver bullet, pointing to the lack of effect of IMNCI training and coverage on clinical outcomes in some settings [17]. Relatedly, a Cochrane systematic review has noted that current evidence regarding IMNCI impact on under-five mortality is of low certainty [12].

Against this background, the primary goal of the current study was to contribute to the emerging body of literature on this topic using nationally representative data from a context that has heretofore been understudied. We examined IMNCI’s effect on neonatal mortality, infant mortality, and—as a mechanism—institutional deliveries in Zimbabwe, where 32 children per 1000 live births die within 28 days and 43 per 1000 live births do not see their first birthday. We also explored the heterogeneous association between IMNCI strategy and place of residence and mother’s level of education. We do so because the previous literature has identified these socio-economic factors as some of the key predictors of child survival [18]. Although the findings enable us to provide policy directions primarily in Zimbabwe, they also offer important lessons for other countries in the region.

## Methods

### Study setting

In Zimbabwe, the first two pillars (see above) of IMNCI were first piloted in four districts (Chegutu, Chipinge, Hwange, and Zaka) in 2002. In the first pillar, the government rolled out in-service training for health workers through a series of eleven-day and six-day (abridged) IMCI courses. Furthermore, nursing school’s curricula were updated to include IMCI modules as part of pre-service training. Provincial and district supervisors were trained and provided resources to conduct IMCI follow up and supervision. As of 2007, a total of 920 health workers (in-service) and 2,502 primary care nurses (pre-service) had been trained [19]. Subsequently, additional on-job trainings and refresher courses were provided in line with the strategy’s reorientation to focus on neonatal deaths in 2012.

As part of strengthening essential drugs and equipment supply (second pillar), IMCI was added into the Essential Drugs List of Zimbabwe (EDLIZ) to ensure adequate supply of childhood diseases management supplies in health facilities—including medicines such as zinc and low osmolarity oral rehydration salts (ORS) for management of diarrhoea [20]. Given the high prevalence of HIV/AIDS, IMCI guidelines were adapted to include management of HIV/AIDS, and paediatric HIV protocols were developed.

In 2004, a training of community-based health workers in four additional districts was conducted (pillar 3), using an approach called the Triple A/CCCD (Assessment, Analysis and Action/ Community Centered Capacity Development). The approach focused on building community capacity to assess problems, analyze their causes, and developing action plans to mitigate the identified problems [21]. This was complemented by recruitment and training of community-based counsellors and childcare committees that provided support to orphans and vulnerable children [22]. By 2007, IMCI strategy was operating in 8 out of the country’s 61 districts at the time.

Following the efforts in other LMICs, in 2010, the Zimbabwean government augmented the strategy to focus on the management of illnesses that are more common among neonates. This reoriented IMNCI strategy focused on newborn care, neonatal conditions including jaundice, gonococcal eye infection, pediatric HIV, nutrition, and malaria. With the support from the Maternal and Child Health Integrated Program (MCHIP), an international non-governmental organisation that works on sustainable improvements in maternal, newborn, and child health, IMNCI strategy was rolled out countrywide in 2012 [23].

### Data source and study design

We used cross-sectional data from the 2015 Zimbabwe Demographic and Health Survey (ZDHS). The ZDHS collects data approximately every five years from a nationally representative sample of households. Women aged 15–49 who were either permanent residents of the selected households or visitors who stayed in the household the night before the survey were eligible for the survey [24]. Specifically, we used the Kids Recode (KR) file available from the DHS website, which had information on all children born between 2010 and 2015 from the interviewed women. The dataset also contained information on women’s characteristics, including age, highest level of schooling completed, and household attributes including access to electricity, source of drinking water, and type of toilet facilities. It also contained information on the wealth quintile to which the household belonged. The ZDHS also calculates a wealth index based on the household’s ownership of selected assets, such as televisions and bicycles, and type of roof and floors, and categorizes households into five quintiles based on the index [25].

### Study variables and statistical approach

Consistent with the primary goals of IMNCI, our key outcome was a binary measure of neonatal mortality. This was defined as the death of a baby before reaching 28 days (one month) of age. This variable was dichotomized into 1 (for neonates who died within a month) and 0 (for neonates who survived the first month of life). In the ZDHS, women were asked the age of their children, including the age at death for children who did not survive.

Given IMNCI’s potential to reduce mortality in subsequent months, we also evaluated IMNCI’s impact on infant mortality. Infant mortality was also defined as binary variable (= 1 for children who died within 11 months of birth and 0 otherwise). It is reasonable to expect IMNCI to influence intermediate outcomes, such as institutional deliveries, particularly through the third pillar [12, 26]. Health facility-based intra-partum care has been shown to improve the chances of survival for newborns, making institutional delivery an important mechanism through which IMNCI can affect neonatal and infant mortality [27, 28]. Therefore, we also evaluated the association between IMNCI and a binary measure of institutional birth (= 1 if the child was delivered at a health institution and 0 otherwise).

Our empirical strategy involved comparing these outcomes for children born within the same geographic cluster—the sampling unit used by ZDHS—before and after the implementation of IMNCI. In the ZDHS, there were 399 clusters, marked using wards, the lowest administrative boundaries. We considered children born in 2010, 2011 and 2012 as born in the pre period and those born in subsequent years as born in the post period.

For each outcome, we estimated adjusted odds ratios from a logistic regression of the following form:

$$Log\;\left(P_{ijt}/1‐P_{ijt}\right)=\alpha +\rho \;IMNCI_{t}+X^{’}{}_{ij}\beta +\lambda_{j}+\varepsilon_{ijt}.$$ (1)

In this equation, P *p*_*ijt*_ = P (Y = 1) represented the probability of death for child *i*, born in cluster *j*, in year *t*. IMNCI_*t*_ was a binary variable (= 1 if the child was born after 2012 and 0 otherwise). X_*jt*_ represented various characteristics of the child, mother, and the household measured at the time of the survey. These characteristics included birth month, gender and birth order of the child, mother’s age and her education level, and wealth quintile for the household. λ_*j*_ were the geographic cluster fixed effects intended to capture confounding due to time-invariant characteristics of the cluster (e.g., geographic remoteness). ε_*ijt*_ was the usual error term. The coefficient *ρ* reflected the association between the outcomes and exposure to IMNCI.

In order to confirm that our results were not solely due to overall trend in outcomes, we conducted similar analyses using two previous waves of the ZDHS data—as falsification tests. The 2005 ZDHS covered births between 2000 and 2005 and the 2010 ZDHS covered births between 2005–2010. In both cases, we imposed a hypothetical intervention date (2002 and 2007, respectively, which are the midpoints of the birth years in the respective datasets) and estimated equations similar to the one above.

The data used in the analysis are publicly available at https://dhsprogram.com/Data/. All data were fully anonymized before we accessed them in accordance with the DHS privacy and data confidentiality policy. We provide Stata codes used to clean the necessary data files and to conduct the analysis in S1 Code.

## Results

In Table 1, we present descriptive statistics of the analytic sample, separately for before and after the implementation of the IMNCI strategy. The sample consisted of 5869 children. The probabilities of neonatal and infant death were 0.03 and 0.05, respectively (which translate to 30 and 50 deaths per 1000 births). Nearly 80% of births took place in health facilities (institutional deliveries). There were similar proportions of boys and girls in the sample, and roughly 28% of children were first born. Approximately, 70% of the mothers had attained secondary level of education or higher. Eighty percent of the children came from households with access to improved and safe drinking water, and 74.1% had access to improved toilet facilities. Urban residents constituted approximately 40% of the total sample.

**Table 1:** Descriptive statistics for the analytic sample (N = 5869).

	*Overall*	*Pre-IMNCI*	*Post-IMNCI*	*P-value*
Neonatal mortality (binary)	0.03	0.03	0.02	0.07
Infant mortality (binary)	0.05	0.05	0.04	0.01
Institutional births (%)	79.7	76.2	82.6	<0.001
Proportion of girls	50.7	51.3	50.2	0.29
*Birth order (%)*				
First	26.9	26.2	27.4	0.58
Second	25.4	25.4	25.4	
Third	20.5	20.9	20.1	
Fourth & above	27.2	27.5	27.1	
Mother’s age (years)	28.7	30.1	27.5	<0.001
*Mother education (%)*				
Primary	30.5	30.7	30.4	0.80
Secondary	69.5	69.3	69.6	
*Source of drinking water (%)*				
Improved	79.9	78.9	79.9	0.85
Not improved	20.1	20.2	20.1	
*Sanitation type (%)*				
Improved	74.1	75.3	73.1	0.05
Not improved	25.9	24.7	26.9	
*Wealth quintile*				
Q1 (poorest)	20.7	19.7	21.5	0.001
Q2	17.5	16.2	18.4	
Q3	15.4	15.3	15.7	
Q4	26.2	26.4	25.9	
Q5 (richest)	20.1	22.4	18.5	
Proportion of urban residents	37.8	40.1	36.0	0.002
N	5869	2697	3172	

Notes: This table shows the descriptive statistics for the analytic sample before and after IMNCI strategy implementation. The *p-values* are from the comparison of pre- and post-implementation values (using student-t test for means and chi-squared test for proportions and distributions).

Comparing before and after the strategy implementation, descriptively, we found that both neonatal and infant mortality were significantly lower in the post-IMNCI period. Relatedly, institutional deliveries were significantly higher in the post period than in the pre period. There was no difference in the composition of birth order, mother’s level of education and access to improved water and sanitation before and after IMNCI. However, mothers were slightly younger and disproportionately from poor households in the post-IMNCI period.

Table 2 presents results from the regressions of the three outcomes on IMNCI. Overall, after controlling for cluster fixed effects and a full set of covariates that included child, mother, and household characteristics, exposure to IMNCI was strongly associated with reductions in both neonatal and infant mortality, and an increase in institutional deliveries. Specifically, exposure to IMNCI was associated with lower odds of neonatal deaths (adjusted odds ratio (95% CI): 0.70 (0.50, 0.98)) and lower odds of infant mortality (adjusted odds ratio (95% CI): 0.69 (0.54, 0.91)). The odds of giving birth in a health facility were approximately twice as high after IMNCI than before (adjusted odds ratio (95% CI): 1.95 (1.67, 2.28)).

**Table 2:** Odds ratio from a logistic regression of neonatal mortality, infant mortality, and in institutional deliveries on IMNCI strategy in Zimbabwe, 2010–2015.

	Neonatal mortality	Infant mortality	Institutional delivery
IMNCI (Binary)	0.70**	0.69***	1.95***
	[0.50,0.98]	[0.54,0.91]	[1.67,2.28]
Child Sex (girl)	0.74*	0.84	1.00
	[0.53,1.03]	[0.66,1.07]	[0.87,1.1]
Birth order (*reference = 1*)			
2	0.54**	0.69**	0.64***
	[0.33,0.91]	[0.48,0.99]	[0.50,0.81]
3	0.77	0.67*	0.49***
	[0.43,1.32]	[0.43,1.03]	[0.37,0.64]
4+	0.89	0.79	0.32***
	[0.46,1.72]	[0.48,1.31]	[0.23,0.43]
Mother’s age (years)	1.00	1.01	1.04***
	[0.96,1.03]	[0.97,1.03]	[1.02,1.06]
Mother’s education (*reference = Primary & less*)			
Secondary & higher	0.85	0.67***	1.60***
	[0.58,1.23]	[0.51,0.89]	[1.35,1.89]
Wealth quintile (*reference = Poorest*)			
Poorer	1.01	0.96	1.25**
	[0.64,1.89]	[0.64,1.43]	[1.00,1.55]
Middle	1.50	1.52*	1.83***
	[0.82,2.73]	[0.98,2.34]	[1418,2.39]
Richer	1.37	1.51	3.80***
	[0.72,2.60]	[0.72,1.84]	[2.79,5.17]
Richest	1.37	1.15	6.01***
	[0.66,2.75]	[0.58,1.68]	[4.60,10.06]
Water (*reference = Not improved*)			
Improved	0.57**	0.59***	0.92
	[0.36,0.90]	[0.42,0.84]	[0.75,1.15]
Sanitation (*reference = Not improved*)			
Improved	0.90	1.28	1.22*
	[0.58,1.40]	[0.91, 1.79]	[0.99,1.49]
N	5,869	5,869	5,869

Notes

* p<0.10

** p<0.05

*** p<0.01. This table shows adjusted odds ratios and 95% confidence intervals (in brackets) from estimating a logistic regression of neonatal mortality, infant mortality, and institutional deliveries (separately) on IMNCI. All models include cluster fixed effects. The regressions are modelled on a full set of covariates which include child characteristics: age, birth month, birth order, gender; mother characteristics: age, education level categorized as primary and less, and secondary and above; and household characteristics capturing whether the household has access to improved drinking water sources and improved sanitation facilities as measured by WHO standards, and the household’s wealth quintile.

We found substantial heterogeneity in the association between IMNCI exposure and measures of mortality across geographic areas (urban vs rural) and mother’s education levels (Table 3). More specifically, exposure to IMNCI was marginally associated with lower odds of neonatal deaths in rural areas (adjusted odds ratio (95% CI): 0.65 (0.42, 0.98)) but not in urban areas (adjusted odds ratio (95% CI): 0.81 (0.45, 1.45)). This was true for infant mortality as well (adjusted odds ratio (95% CI): 0.70 (0.51, 0.96) for rural versus adjusted odds ratio (95% CI): 0.68 (0.43, 1.08) for urban). However, exposure to IMNCI was associated with higher odds of institutional deliveries in both urban (adjusted odds ratio (95% CI): 2.10 (1.45, 3.04)) and rural areas (adjusted odds ratio (95% CI): 1.94 (1.63, 2.30)).

**Table 3:** Odds ratio from a logistic regression of neonatal mortality, infant mortality, and institutional deliveries on IMNCI strategy in Zimbabwe, 2010–2015, by urbanicity and mother’s education level.

	Neonatal mortality		Infant mortality		Institutional deliveries	
*Panel A*. *Urban vs Rural*						
	Urban	Rural	Urban	Rural	Urban	Rural
IMNCI	0.81	0.65**	0.68	0.70**	2.10***	1.94***
	[0.45,1.45]	[0.42,0.98]	[0.43,1.08]	[0.51,0.96]	[1.45,3.04]	[1.63,2.30]
N	2,130	3,592	2,221	3,648	2,221	3,648
*Panel B*. *Educational level*						
	Primary	Higher	Primary	Higher	Primary	Higher
IMNCI	0.96	0.59**	0.96	0.57***	1.99***	2.10***
	[0.53,1.73]	[0.39,0.92]	[0.63,1.46]	[0.41,0.80]	[1.47,2.45]	[1.70,2.60]
N	1,790	4,079	1,790	4,079	1,790	4,079

Notes

*p<0.10

** p<0.05

*** p<0.01. This table shows adjusted odds ratios and 95% confidence intervals (in brackets) from estimating a logistic regression of neonatal mortality, infant mortality, and institutional deliveries (separately) on IMNCI, separately for urban vs rural (Panel A) and mother’s education level (Panel B). All models include cluster fixed effects. The regressions are modelled on a full set of covariates which include child characteristics: age, birth month, birth order, gender; mother characteristics: age, education level categorized as primary and less, and secondary and above; and household characteristics capturing whether the household has access to improved drinking water sources and improved sanitation facilities as measured by WHO standards, and the household’s wealth quintile.

Across the mother’s education gradient, the exposure to IMNCI was associated with lower odds of both neonatal deaths (adjusted odds ratio (95% CI): 0.59 (0.39, 0.92)) and infant deaths (adjusted odds ratio (95% CI): 0.57 (0.41, 0.80)) for mothers with higher education. Conversely, for mothers with primary education or lower, exposure to IMNCI was not associated with neonatal (adjusted odds ratio (95% CI): 0.96 (0.53, 1.73)) or infant deaths (adjusted odds ratio (95% CI): 0.96 (0.63, 1.46). Similar to the case of urban versus rural, IMNCI exposure was associated with higher odds of institutional deliveries for mothers with higher education (adjusted odds ratio (95% CI):2.10 (1.70, 2.61) as well as mothers with primary education or lower (adjusted odds ratio (95% CI): 1.99 (1.47, 2.45)).

As mentioned above, to assess the robustness of our results, we performed analyses similar to the main analysis but using previous rounds of the ZDHS data and by imposing a hypothetical intervention date. If we found the association between the hypothetical intervention and the outcomes to be similar to the main results, our confidence in the main analysis would be reduced substantially. Such a finding would indicate a downward trend in neonatal and infant mortality even before the commencement of IMNCI. Reassuringly, the results presented in Table 4 generally show increased odds for both child mortality outcomes and lowered odds of institutional deliveries using the pseudo pre- and post-strategy periods. These findings suggest that our main results above are unlikely to be driven by overall trends alone.

**Table 4:** Falsification and robustness checks using previous rounds of the demographic and health surveys.

	Neonatal mortality	Infant mortality	Institutional deliveries
*Panel A*. *Using ZDHS 2005*			
IMNCI	1.50*	1.10	0.83**
	[1.00,2.25]	[0.86,1.42]	[0.71,1.00]
N	5,096	5,096	50,96
*Panel B*. *Using ZDHS 2010*			
IMNCI	1.75***	1.20	0.94
	[1.17,2.62]	[0.91,1.58]	[0.81,1.08]
N	5,390	5,390	5,390

## Discussion and conclusion

Without a significant change in the current trajectory of mortality rates, Zimbabwe is unlikely to meet the SDG target on reducing neonatal deaths to 12 per 1000 births by 2030. This study evaluated the association between exposure to IMNCI and neonatal and infant mortality, as well as institutional deliveries in the context of Zimbabwe. While previous studies have examined IMNCI in Asia and North Africa [8, 12], to our knowledge, this is the first study to evaluate its effects on mortality in sub-Saharan Africa. Among the few studies that have assessed IMNCI’s effect on mortality directly, a study from India has found that IMNCI significantly reduced neonatal and infant mortality [16]. Another study, from Indonesia, has reported similar effects on overall mortality occuring between seven days since birth and five years of age [14]. Our findings are in agreement with those studies. Our findings suggest that IMNCI may have also had positive spillover on the quality of care received by older children, as reflected by the improvement in infant mortality which was not the primary the focus of the reoriented IMNCI strategy.

An understanding of the pathways through which IMNCI improved child survival is important for further refinement of the strategy. Our results suggests that a rise in institutional deliveries is an important mechanism through which IMNCI may have helped reduce neonatal and infant mortality in Zimbabwe. Previous research shows that institutional delivery lowers neonatal deaths mainly through improved newborn practice, including timely initiation of breastfeeding, exclusive breastfeeding, delayed bathing, and appropriate cord care [12, 16]. In examining the heterogenous association between IMNCI and the outcomes based on mother’s education and urban versus rural status, we found that IMNCI performed better among more educated mothers and those in rural areas.

Our findings should be understood in light of a number of caveats. First, the cross-sectional nature of our data does not allow us to establish that our estimates are causal, although we have adjusted for a wide range of potential confounders on which data were available. Second, the data were collected exclusively from mothers and might be prone to recall bias, although the possibility of such bias are lower for mortality (it would be uncommon for mothers to not remember infant or neonatal deaths incorrectly) than for institutional deliveries. Third, we are unable to account for factors that can vary within a cluster over time, such as changes in income, which can affect survival rates independent of IMNCI. Finally, the sample of children and mothers in the post-IMNCI period differed in notable ways from the sample in the pre-IMNCI period (Table 1). Specifically, individuals in the post-IMNCI period were more likely to be poorer and rural. As shown in S1 Table, before IMNCI, poorer and rural households had similar neonatal and infant mortality rates compared to their richer and urban counterparts. However, poorer and rural households had lower rates of institutional deliveries, implying that there was a greater room for improvement among these households. Therefore, some of the improvement in institutional deliveries among these households post-IMNCI relative to their richer and urban counterparts could be due to the lower baseline (pre-IMNCI) rates.

It is also important to understand these encouraging findings within the broader context of Zimbabwe’s socio-political climate. The Zimbabwean health delivery system is largely public sector driven, complemented by church-run hospitals. The private health sector is small and reserved mainly for the elite in urban centers [29, 30]. In the early 2000s, Zimbabwe endured years of socio-economic and political upheaval, characterized by hyperinflation, massive health worker brain-drain, and political instability [29, 31, 32]. The instability put a major strain on the health infrastructure built since Zimbabwe’s independence in 1980. The 2008 cholera pandemic that claimed over 5 000 lives further eroded confidence in the health care system [33]. However, the political coalition that came to power in 2009 provided a temporary reprieve from the political and economic flux seen in the early 2000s. IMNCI was implemented in a relatively calm and stable period in the aftermath of these instabilities, marked by notable improvements in health investments.

There are a number of other related factors that likely contributed to IMNCI’s success, and these factors may offer lessons for other countries aiming to reduce mortality though IMNCI. First, IMNCI might have benefitted from the results-based financing (RBF) program that was adopted in 2011. As a part of this program, the government provided cash and in-kind incentives to health facilities, health providers, managers, and consumers as an incentive to utilize or deliver maternal and child health care services. These incentives bolstered health worker motivation, promoted retention of critical human workforce such as midwives, and improved healthcare utilization in general [34]. The RBF efforts were augmented by the operationalization of the National Human Resources for Health Policy which facilitated optimum production, training, management and retention of health workers in the public health sector [35]. Specifically, the policy introduced a “task shifting” initiative in which Primary Care Nurses were upskilled to provide basic MNCH services [36]. The task shifting initiative may have benefitted the healthcare workforce strengthening component of IMNCI (under pillar 1).

Another related program, called the Health Transition Fund (HTF), may have further facilitated IMNCI. The HTF, managed by UNICEF, was a multi-donor pooled fund, which aimed to support the government of Zimbabwe to “achieving the highest possible level of health and quality of life for all Zimbabweans”(37 p7). With HTF, the government eliminated maternity user fees in government health facilities, and increased the proportion of facilities having all essential medicines from national average of 12.1% in 2011 to 85.4% in 2012 [37, 38].

Third, as part of the RBF program to strengthen health governance, the Ministry of Health and Child Care (MOHCC) revived the health centre committees (HCCs). The HCCs consist of representatives from the local area and the health facilities [39]. The existence of these committees provided an opportunity for local participation in the day-to-day functioning and planning activities of the health centres, and promoted accountability. Indeed, health facilities with HCCs have been documented to improve client satisfaction [34].

Finally, IMNCI came with a consolidated childhood illnesses register that facilitated a systematic and harmonized assessment, classification, and treatment of young children [23]. Efficient health information systems are a vital component to timely and appropriate clinical decision making and consequently better outcomes [40].

These factors also help explain the differential effect that IMNCI had on urban and rural areas. For example, most funding from the HTF was sent to rural health facilities. These funds facilitated, amongst other interventions, the construction of “waiting mothers shelters”—waiting homes near the health facility where women in their third trimesters could wait and be closely monitored by health staff. By tackling geography-related barriers (distance, poor roads, transportation cost) head-on, these facilities improved access and utilization of antenatal and postnatal care services significantly [41].

Additional research is needed to understand whether the current pace in reducing neonatal and infant mortality will sustain in Zimbabwe. Zimbabwe’s health budget is still heavily depended on donor support, which also threatens the sustainability of recent gains in neonatal and infant mortality. In 2018, the budget allocated to health was 8.3% of the national budget, more than half of which was provided by development partners [42]. While the allocation is an improvement from previous years, it falls far short of the 15% allocation to the health sector—a commitment that countries including Zimbabwe made in the Abuja declaration [36]. Sustaining the positive effect of IMNCI and meeting the SDGs targets will require continued political will in raising investment in health and perhaps widening the role of the small private sector that has so far been limited to a few urban centres [29, 30].

Future research can also explicate the specific causes of mortality that IMNCI strategy affected. In some countries DHS collects data on verbal autopsy (VA) and social autopsy (SA). These tools are used to identify specific causes of death in settings with limited record keeping and to better understand modifiable cultural, social, and health system factors affecting health care access and utilization [43]. Unfortunately, such data are not yet available for Zimbabwe. An understanding of mortality causes using VA and SA data can help identify additional areas for programmatic and policy improvements.

## Supporting information

S1 Table Supplemental table. (DOCX)

S1 Code Stata codes used to clean the data and conduct the main analysis. (DO)
