# Supplementary material for: Integrated management of neonatal and childhood illness strategy in Zimbabwe: An evaluation
Source: PLOS Glob Public Health. 2021 Nov 22;1(11):e0000046. doi: 10.1371/journal.pgph.0000046 (PMC10021544; doi:10.1371/journal.pgph.0000046)
Supplement: S1 Table — (DOCX) [file pgph.0000046.s001.docx]

| **S1_Table. Descriptive statistics on neonatal mortality, infant mortality, and institutional deliveries by wealth quintile and urbanicity before IMNCI implementation** | | | | | | | |
| --- | --- | --- | --- | --- | --- | --- | --- |
|  | Poorest | Poorer | Middle | Richer | Richest | *P-values* |  |
|  |  |  |  |  |  |  |  |
| Neonate | 0.03 | 0.03 | 0.04 | 0.03 | 0.03 | 0.83 |  |
| Infant | 0.06 | 0.05 | 0.07 | 0.05 | 0.04 | 0.41 |  |
| Delivery | 0.55 | 0.62 | 0.73 | 0.88 | 0.93 | <0.001 |  |
| N | 530 | 438 | 413 | 713 | 603 |  |  |
|  |  |  |  |  |  |  |  |
|  | Rural | Urban |  |  |  | *P- values* |  |
| Neonate | 0.03 | 0.03 |  |  |  | 0.27 |  |
| Infant | 0.06 | 0.05 |  |  |  | 0.08 |  |
| Delivery | 0.66 | 0.92 |  |  |  | <0.001 |  |
| N | 1618 | 1079 |  |  |  |  |  |

Notes: This table shows the descriptive statistics for the analytic sample before IMNCI strategy implementation. The p-values are from the comparison of proportions across the groups (i.e., between wealth quintiles and rural vs urban). For wealth quintiles, the p-values are from a joint orthogonality test (i.e., the null hypothesis is that none of the groups are different from each other; if p<0.05, it means that at least two sets of proportions are different from each other at the five percent significance level).
